# Engineering Protein–Polyelectrolyte Interactions for Cellular Applications

**DOI:** 10.1146/annurev-chembioeng-100722-105929

**Published:** 2025-03-13

**Authors:** Rachel S. Fisher, Jane Liao, So Yeon Ahn, Nisha Modi, Aaron K. Kidane, Allie C. Obermeyer

**Affiliations:** 1 Chemical Engineering, Columbia University, New York, NY, USA; 2 Cellular and Molecular Physiology and Biophysics, Columbia University, New York, NY, USA

**Keywords:** protein engineering, polyelectrolyte, complex coacervation, biomolecular condensates, polyelectrolyte complex micelles

## Abstract

Protein–polyelectrolyte interactions are fundamental interactions in biology that occur at every length scale, from protein–DNA complexes to phase-separated organelles. They drive processes ranging from gene transcription and DNA synthesis to viral assembly. Protein engineering is a powerful way to modulate these interactions, both to probe endogenous function and to engineer novel interactions between species. In this review, we consider the various noncovalent interactions that govern the formation and behavior of these complexes, and we discuss how protein modifications such as changes to structure, charge, and charge patterning affect them. We highlight recent examples where engineering changes to protein–polyelectrolyte interactions have helped elucidate biological function, and we then focus on recent efforts toward de novo material design of synthetic biomolecular condensates and functional nanoassemblies.

## 1. INTRODUCTION

**Figure 1:**
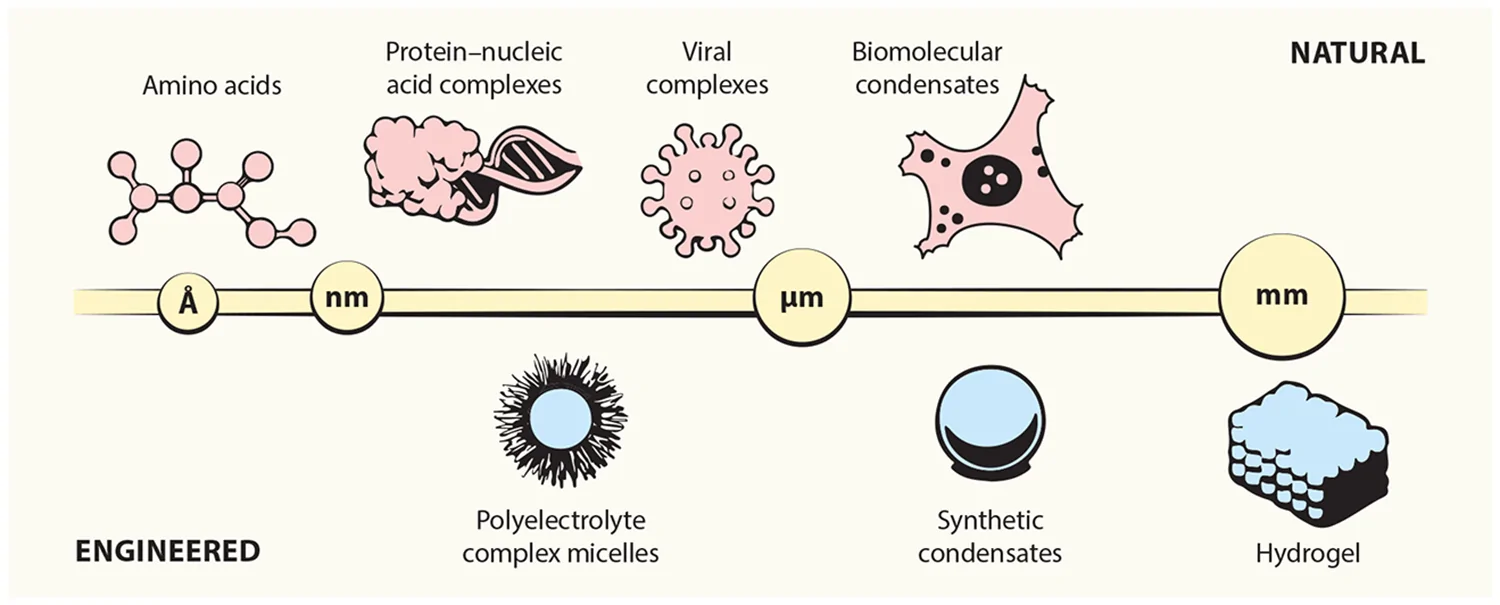
Range of length scales found in (*top*) natural and (*bottom*) engineered protein–polyelectrolyte interactions.

Protein–polyelectrolyte (PE) interactions are fundamental biological interactions that occur at every length scale within a cell (Figure 1). At the molecular scale, individual proteins bind to DNA, where, for example, DNA synthesis is mediated by the interaction between DNA and DNA polymerase. At the nanoscale, coat proteins assemble around viral genomes to form capsids, creating self-assembled viral particles (1). At the micrometer scale, proteins phase separate with RNA to create membraneless organelles, which are dynamic assemblies of tens to hundreds of components held together by a delicate balance of multivalent interactions. Understanding the nature of these important biological interactions and the interplay between proteins and biological PEs can provide a wealth of information about biological processes at every scale, but the very nature of these interactions and the fact that they can lead to large microscale assemblies make it challenging to study them.

**Polyelectrolyte (PE):**

a polymer or macromolecule composed entirely or partly of either ionic or ionizable groups; examples include nucleic acids, proteins, and polysaccharides

Protein engineering provides the opportunity to edit the primary amino acid sequence of a protein. Although more commonly used to probe protein structure and function, protein engineering enables thoughtful changes at multiple levels to probe the interactions that underlie protein–PE assemblies. These changes can be as large as the deletion of entire domains or as simple as a single-amino-acid mutation. By truncating proteins or systematically removing whole domains, one can map the role of different regions of a protein in driving PE interactions. For example, a study of different combinations of domains found that the disordered hinge region of heterochromatin protein 1α, which is essential for heterochromatin formation and maintenance, is sufficient for DNA compaction and phase separation with DNA (2).

Protein engineering can also be used to rearrange residues to assess the impact of amino acid patterning or to alter the composition of proteins in order to identify the effect of a specific type of noncovalent interaction on the interactions with PEs (3). For example, mutating all charged surface residues to neutral allows one to probe the role of ionic interactions, and a simple swap of the cationic residues lysine and arginine can be used to probe the role of cation–*π* interactions. Finally, point mutations can be used to selectively alter residues at key locations. These small modifications can result in changes to protein structure, which, in turn, can influence a protein–PE interaction or directly alter the selectivity of binding at a recognition site.

**Protein engineering:**

creation or modification of a protein sequence, usually achieved by editing the DNA sequence of the encoding gene

Beyond probing native interactions, protein engineering can be used to create proteins with new functionality. In a parallel to nature, at length scales ranging from the molecular to the micrometer scale, efforts are underway to design or control protein–PE interactions so as to create de novo materials for biomedical and biotechnological applications. At the scale of individual protein–PE complexes, DNA-cleaving nucleases used in genome-editing applications can be mutated to alter their cleavage specificity, enabling the recognition of new genomic targets (4). At the nanoscale, increasing the surface charge, or supercharging, can be used to introduce electrostatic interactions, thereby promoting coassembly with polymers and the formation of PE core micelles that can be used to deliver protein therapeutics (5-8). At the micrometer scale, proteins have been multimerized or mutated to create synthetic biomolecular condensates that act as cellular hubs to recruit specific biomacromolecules and promote biocatalysis (Figure 1).

**Biomolecular condensates:**

membraneless compartments in cells with concentrated biomolecules

The goal of this review is to take stock of areas where protein engineering is being used to modulate protein–PE interactions for applications in cellular delivery and cell-based technologies. We discuss two broad areas where exciting recent advances have been made. The first is the understanding and engineering of biomolecular condensates, and the second is the design and application of nanoscale assemblies. In both areas, protein engineering is being used in innovative ways to manipulate PE–protein interactions, with the goal of probing endogenous function and engineering novel interactions between species. In general, the biomolecular condensate field has recently turned from studying natural systems to controlling these systems or creating synthetic mimetics. Conversely, the design of nanoparticles traditionally used polymeric materials, but recent efforts have sought to replace synthetic polymers with more biocompatible engineered proteins. By reviewing these areas side by side, we hope to highlight both the similarities between them and the potential convergence they are undergoing.

In Section 2, we present an overview of the noncovalent interactions underlying all protein–PE complexation. Most relevant to protein engineering applications is the behavior of individual amino acids; therefore, we focus on the nature and strength of the noncovalent interactions of each amino acid. In Section 3, we describe cases where protein–PE interactions lead to the formation of biomolecular condensates, then discuss the use of protein engineering to modify these interactions and thereby elucidate or regulate their behavior. In Section 4, we review the design and modification of synthetic condensates, as informed by the discoveries discussed in Section 3. In Section 5, we move down in scale and focus on protein–PE nanoassemblies as tools for intracellular delivery, in light of efforts to engineer protein cargo and proteins as materials for micelles. Finally, we look ahead to identify emerging areas of interest in the engineering of protein–PE interactions.

**Synthetic condensates:**

models of endogenous biomolecular condensates using unnatural or natural biomolecules

## 2. NONCOVALENT INTERACTIONS DRIVE PROTEIN–POLYELECTROLYTE INTERACTIONS

### 2.1. Noncovalent Interactions Mediating Protein–Polyelectrolyte Assemblies

The transient and dynamic nature of protein–PE assemblies is due largely to the weak, noncovalent interactions that drive their formation. These noncovalent interactions typically provide a few kilocalories per mole of enthalpic stabilization (~1–5 kcal/mol or 1.7–8.3 *RT*, where *R* is the gas constant and *T* is temperature), but when many such interactions work cooperatively they can create stable complexes from the nano- to the macroscale, as observed in the formation of folded proteins, protein aggregates, and crystal lattices (9). Critically, the relatively weak nature of noncovalent interactions enables dynamics, rearrangement, and even dissolution of protein–PE complexes on biologically relevant timescales. For example, in response to stress, endogenously phase-separating proteins such as fused in sarcoma, heterogeneous ribonucleoprotein A1, and the RNA-dependent DEAD-box ATPases (DDX) orchestrate the sequestration of mRNAs into membraneless organelles (10, 11)—a reversible process facilitated by numerous noncovalent interactions that contribute to favorable demixing of protein and RNA biomolecules. Despite the weak noncovalent interactions that commonly occur in disordered biopolymers, they can still deliver strikingly high specificity and affinity in the complexes they form (12-15). For transcription factors, which are enriched in intrinsically disordered regions (IDRs) (16), long IDRs play a significant role in directing their localization to binding sites along the genome and contribute to specificity through direct binding with genomic DNA adjacent to regulatory motifs (17, 18). The reliable control of noncovalent interactions to form protein assemblies with a range of biological PEs has emerged following billions of years of evolution (19). Recent efforts to engineer tunable protein–PE interactions have sought to mimic the specificity, strength, and dynamics found in their natural counterparts. Below, we discuss the range of noncovalent interactions that can be engineered in proteins and how they influence the enthalpy and/or entropy of interactions with PEs.

**Figure 3:**
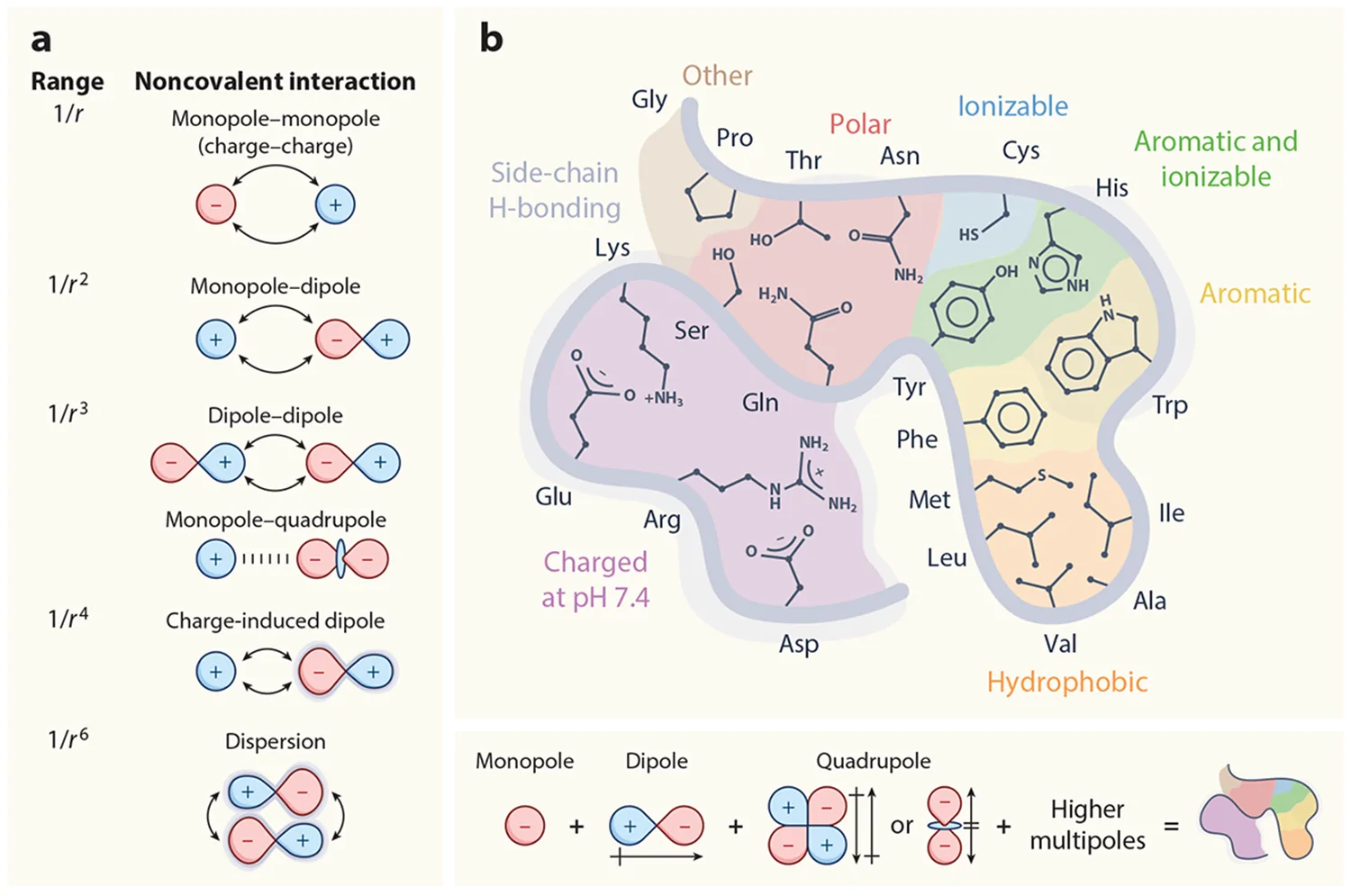
(*a*) Length scales of noncovalent interactions that commonly occur between proteins and polyelectrolytes and schematic representations of these interactions. (*b*) The 20 native amino acids can be classified by the primary noncovalent interactions that they engage in, but note that these noncovalent interactions are ultimately the sum of all electronic multiple moments.

In this review, we use the term noncovalent interactions to refer to interactions between any of the following: charged, polar, quadrupolar, and nonpolar (i.e., hydrophobic) species (Figure 3). In the simplest framework, interactions that drive protein and PE complexation can be classified mainly as those with favorable enthalpy due to electrostatic interactions between charges or partial charges and those with favorable entropy due to the release of relatively structured waters and small ions. Thus, charge–charge interactions as well as hydrogen-bonding and cation–π interactions can all be understood through the lens of favorable electrostatic interactions. These noncovalent interactions with favorable enthalpy can be coupled to or in opposition with favorable entropy that occurs upon the burial of hydrophobic moieties.

The strength of noncovalent interactions driven by electrostatics follows from Coulomb’s law and depends on both the moment of the charge distribution and the distance between species. For charged species (or monopoles), the interaction energy is proportional to the product of each of the charges and decreases with distance as 1*/r*. While attractive interactions between oppositely charged species are quite large in a vacuum (>60 kcal/mol at 5 Å separation), the high dielectric constant of water (~74 at 37°C) (20) effectively attenuates the coulombic attraction between charged species, decreasing the overall favorability of a single electrostatic interaction (9, 21). Note that the context of the medium for charge–charge (or monopole–monopole) interactions is paramount, as a single charge–charge interaction in the core of a protein can contribute 3–4 kcal/mol of stabilization due to the lower dielectric constant in the core of a protein, whereas those on the surface contribute lower stabilization energies (typically 0–2 kcal/mol) (22). For higher moments, such as dipoles or quadrupoles, the interaction decreases more rapidly with distance, typically involves partial charges on the species, and has geometric constraints that result in spatial anisotropy, all of which serve to weaken the overall interaction (Figure 3). However, each of these higher-order noncovalent interactions can still play important roles in protein–PE complexation. For example, the arginine–glycine (RGG/RG) motif, present in more than 1,000 human proteins (23), depends critically on the quadrupolar moment of arginine to stabilize complex formation with mainly RNA but also other proteins (24). The weak nature of these quadrupolar interactions with increasing distance also means that a degenerate specificity can be incurred with slight changes to RGG/RG domain sequences and the spacing of these domains (25, 26). Measurements of the strength of each type of interaction (e.g., dipole–dipole, monopole–quadrupole) come primarily from the protein-folding literature, where specific examples demonstrate stabilization energies in the range of a few kilocalories per mole (27). Importantly, these noncovalent, electrostatic interactions are ultimately the sum of the interactions between all of the electronic multipole moments that are possible between the interacting species, making deconvolution of the energetic contribution of a single type of interaction virtually impossible in a biological setting. For example, noncovalent interactions between arginine and tyrosine involve monopole–quadrupole (as well as dipole–dipole, dipole–quadrupole, etc.) interactions. Even species without permanent multipole moments engage in energetically favorable noncovalent interactions via dispersion forces, wherein fluctuating dipole moments are induced between nearby nonpolar species in so-called London dispersion forces or van der Waals interactions.

In addition to these favorable electrostatic noncovalent interactions, the role of entropy in driving complexation between proteins and PEs cannot be overlooked. We briefly discuss the role of entropy in noncovalent interactions of proteins here, but we refer the reader to more indepth discussions in various physical chemistry or biophysics textbooks (21, 28). Although both proteins and PEs are readily soluble in water, they often contain relatively hydrophobic domains that result in the structuring of water around the hydrophobic area. Binding or complexation between these hydrophobic domains, both intra- and intermolecularly, is favorable largely because of the release of these relatively ordered water molecules into the bulk liquid. This hydrophobic effect is responsible for the formation of phospholipid bilayers, drives protein folding into globular domains, and plays a role in many ligand– or drug–protein binding interactions (27). For protein–PE interactions, it is also important to consider the entropy of bound counterions in addition to any bound water molecules. Because macromolecules are polyionic species, binding between them can be entropically favorable as a result of the release of many counterions (29-32).

### 2.2. Primary Sequences Tune Noncovalent Interaction Strength

The great diversity of amino acid side-chain composition lends itself to a nearly limitless array of proteins with different sequences, structures, and interactions with PEs, among other characteristics. The 20 canonical amino acids alone yield 3,200,000 unique sequence combinations in a polypeptide that is just 5 amino acids long. Yet, proteins have also evolved over billions of years to have numerous consensus sequences that serve similar purposes but have a striking level of specificity due to amino-acid-level sequence variations. This evolutionarily guided sequence design lends itself to the crude design of protein–PE interactions a priori. To engineer proteins and their interactions with PEs, one must appreciate that each of the 20 canonical amino acids can engage in different types of noncovalent interactions. Below, we describe the noncovalent interactions mentioned above and discuss which of the amino acids can engage in such interactions.

As discussed above, electrostatic interactions play a key role in promoting protein–PE interactions. For charge–charge (or monopole–monopole) interactions, there are four amino acids that are natively charged at physiological pH (7.4). Aspartic acid and glutamic acid are negatively charged carboxylates, while lysine and arginine present positively charged amines and guanidiniums, respectively. However, additional residues can be ionized, depending on the local environment. They include histidine (*p*K_a_ ≈ 6), which becomes positively charged upon ionization, and cysteine (*p*K_a_ ≈ 8.2) and tyrosine (*p*K_a_ ≈ 10.1), which can become negatively charged. Additional side chains can contribute charged interactions when posttranslationally modified. The primary examples are serine, threonine, and tyrosine, all of which can be phosphorylated by kinases (33). Note that while charged amino acids can contribute charge–charge interactions, each side chain contributes different higher-moment noncovalent interactions. The impact of these additional noncovalent interactions on protein interactions with biological PEs has been demonstrated most notably for the cationic amino acids lysine and arginine (34-36).

Next we consider interactions between dipoles, the most important of which is arguably the hydrogen bond. All amino acids have backbone carbonyl and amide groups that can form hydrogen bonds. These backbone hydrogen bonds are most frequently found in secondary structural features of proteins, including α-helices and β-sheets. These backbone moieties can also function as hydrogen-bond acceptors or donors with side-chain functional groups and water molecules (27). While all amino acids can form backbone hydrogen bonds, 40% of side chains—glycine, proline, alanine, isoleucine, leucine, methionine, phenylalanine, and valine—lack both hydrogen-bond donors and acceptors. Beyond hydrogen bonds, many amino acid side chains have permanent dipole moments that can engage in dipolar interactions. In addition to those with charged side chains, the polar side chains of serine, threonine, asparagine, and glutamine contain relatively large partial charges that increase the magnitude of the dipole moment.

Additional noncovalent interactions among proteins that play important roles, ranging from the mediation of protein folding to the formation of membraneless organelles, are those involving the π systems of the aromatic amino acids histidine, tyrosine, phenylalanine, and tryptophan. The quadrupole moment of these side chains can engage in favorable π–π interactions either in an edge-to-face orientation or in offset parallel stacks. The aromatic side chains also contribute favorable interactions with cations in so-called cation–π interactions, where the cationic residue has favorable enthalpy when oriented perpendicularly to the plane of an aromatic moiety, in the partially negative π–electron cloud. Favorable anion–π interactions exist as well, where anions preferentially bind to the partially positive edges of the aromatic rings.

Finally, hydrophobic interactions, which include both favorable van der Waals interactions and favorable entropy due to release of structured water molecules, are dominated by the aliphatic side chains of alanine, valine, isoleucine, leucine, methionine, and cysteine. The aromatic side chains of phenylalanine and tryptophan, which are hydrophobic in addition to having quadrupole moments, also contribute to hydrophobic noncovalent interactions. These interactions can play an important role in driving protein assembly, whether that is folding into α-helices, anchoring into the phospholipid membrane, or forming complexes with PEs. For example, in Alzheimer’s pathologies, faulty cleaving of the amyloid precursor protein results in the addition of two hydrophobic residues to the extracellular cleaved product, which drives aggregation of the resulting Ab42 peptide as a result of stronger hydrophobic interactions of the additional isoleucine and alanine residues (37, 38).

The 20 amino acids are typically classified into the following five groups: charged (arginine, histidine, lysine, aspartic acid, glutamic acid), uncharged polar (serine, threonine, asparagine, glutamine), hydrophobic (alanine, isoleucine, leucine, methionine, valine), aromatic (phenylalanine, tryptophan, tyrosine), and special cases (cysteine, glycine, proline) (Figure 3b). However, as discussed above, each group includes a wide range of side-chain compositions that allow amino acids of the same class to have dramatically different noncovalent interactions. Thus, when engineering proteins, it is important to consider the full range of possible noncovalent interactions that various amino acids can engage in. The ability to tune protein–PE interaction strength by modifying protein sequences grants us the ability to engineer specific mesoscale interactions to enable applications ranging from synthetic biology to novel protein drug design to the development of various in vitro and in vivo biomaterials. In the next three sections, we review recent efforts to engineer protein–PE interactions from the meso- to the nanoscale.

## 3. PROTEIN ENGINEERING TO PROBE BIOMOLECULAR CONDENSATES

### 3.1. Role of Protein–Polyelectrolyte Interactions in Biomolecular Condensates

The dynamic multivalent interactions between biopolymers in a cell can lead to the formation of mesoscale assemblies known as biomolecular condensates. Both prokaryotic and eukaryotic cells contain abundant biopolymers such as proteins, nucleic acids, lipids, and carbohydrates, resulting in macromolecular crowding that can drive the phase transition of biopolymers, akin to the thermodynamic phase separation of saturated polymers in solution (39). Above a threshold concentration, such systems will minimize free energy by forming a macromolecule-rich dense phase that coexists with a low-concentration dilute phase. Although the term biomolecular condensate makes no assumptions about physical mechanisms or material states, phase separation has emerged as a unifying principle to explain how biomolecules, such as proteins and RNA, concentrate and self-assemble into a condensed phase. Increasing evidence suggests that biomolecular condensates play intricate roles in various cellular functions, including but not limited to gene regulation, stress response, noise buffering, storage, and cell division (40). The dysregulation of condensates has also been implicated in various diseases such as cancer (41), metabolic disease (42), neurodegenerative disease (43), and viral infection (44).

Notably, numerous assemblies previously described as granules, foci, puncta, or bodies fall under the umbrella term biomolecular condensates. In particular, ribonucleoprotein granules, such as the nucleolus, stress granules (SGs), and neuronal granules, represent an important class of biomolecular condensates that typically contain RNA-binding proteins and RNA (45). This key class of biomolecular condensates highlights the biological significance of protein–PE interactions in governing the assembly, composition, material properties, and cellular functions of a broad swath of condensates.

**Ribonucleoprotein granule:**

RNA–protein assembly that can form through multivalent RNA–RNA, RNA–protein, or protein–protein interactions

**Stress granule (SG):**

biomolecular condensate consisting of RNA and protein that forms in response to various stress conditions by temporarily sequestering and regulating the translation of mRNA

Biomolecular condensates are complex, multicomponent assemblies that can consist of hundreds of distinct biomolecules, so isolating the direct effect of individual protein–PE interactions is not always trivial. Engineering approaches such as heterologous expression, mutagenesis, expansion of repeat motifs, protein fusions, and deletions of relevant protein domains are extensively employed in attempts to elucidate the most relevant parameters of protein and RNA interactions in biomolecular condensates. By systematically manipulating the intrinsic properties of both the protein and PE constituents, we can better understand the underlying interactions governing these dynamic cellular structures as well as their functional implications (Table 1).

### 3.2. Protein and Polyelectrolyte Structures

**Figure 4:**
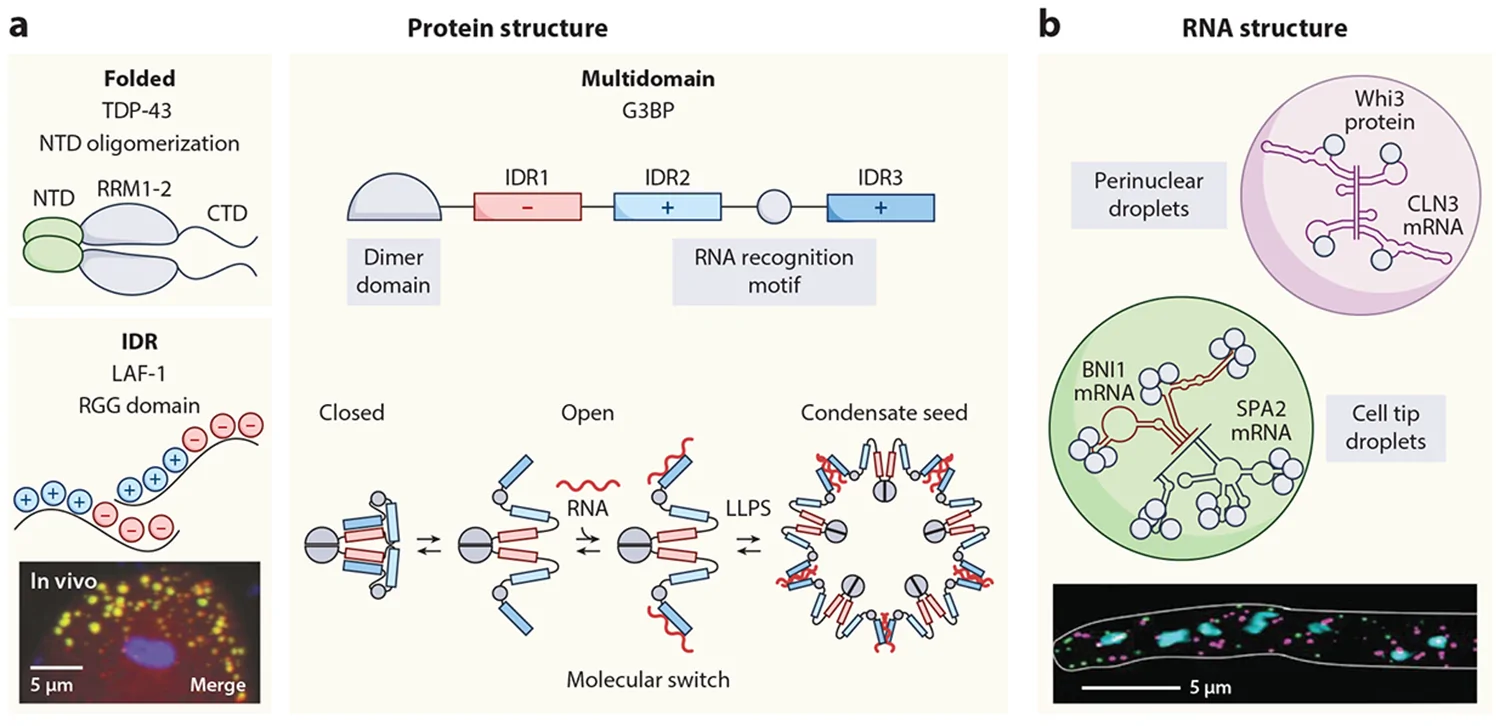
Protein and polyelectrolyte structures. (*a*) TDP-43 contains a folded domain and LAF-1 contains a disordered domain, both of which can be key for phase separation. Multiple domains and conformations fine-tune the formation of condensates with G3BP1. (*b*) RNA structure affects Whi3 condensate behavior. Abbreviations: CTD, C-terminal domain; IDR, intrinsically disordered region; LLPS, liquid–liquid phase separation; NTD, N-terminal domain; TDP-43, TAR DNA-binding protein. Panel *a* adapted with permission from References 46 and 57; copyright National Academy of Sciences. Panel *b* adapted from Reference 47.

The structural features of both proteins and PEs can greatly influence their interaction and the resulting network properties. Many proteins that have been identified as drivers of phase separation in endogenous condensates have a range of structural features, from fully folded domains to IDRs. For example, the TAR DNA-binding protein (TDP-43) found in nuclear bodies and SGs has a folded N-terminal domain that can enhance phase separation upon self-oligomerization (53-56) (Figure 4a). In contrast, IDRs lack a defined structure; often have low sequence complexity; and can be enriched in charged residues such as lysine, arginine, glutamate, and aspartate. For example, the disordered RGG domain of the P-granule protein LAF-1 is necessary and sufficient for both phase separation and RNA–protein interactions (57).

Often, folded domains and IDRs work synergistically in multidomain proteins to modulate phase separation. An example of a multidomain protein is G3BP1, which, together with RNA, forms a core protein–RNA interaction network to drive the formation of SGs in eukaryotic cells (46). Specifically, the dimer domain increases the valency of G3BP1 for RNA, while the folded RNA recognition motif binds RNA. More interestingly, the three distinct IDRs in G3BP1 appear to fine-tune the saturation concentration by forming a molecular switch that responds to an increase in free intracellular RNA and seeds liquid–liquid phase separation (LLPS) and SG assembly.

In addition to the protein structure, the secondary structure of RNA components can impart condensates with distinct biophysical and biological properties. For instance, Langdon et al. (47) showed that the RNA-binding protein Whi3 forms distinct droplets near the nucleus and in the polarized cell tips of the filamentous fungus *Ashbya gossypii*. Interestingly, compositional specificity was conferred from the mRNA structure, particularly from its capacity for homo- or heteromeric interactions. Furthermore, binding of the Whi3 protein altered the conformational dynamics of the different RNA complexes, demonstrating that protein–RNA interactions can further regulate the immiscibility of coexisting condensates in the cell (Figure 4b).

### 3.3. Net Charge, Surface Charge, and Charge Patterning

**Figure 5:**
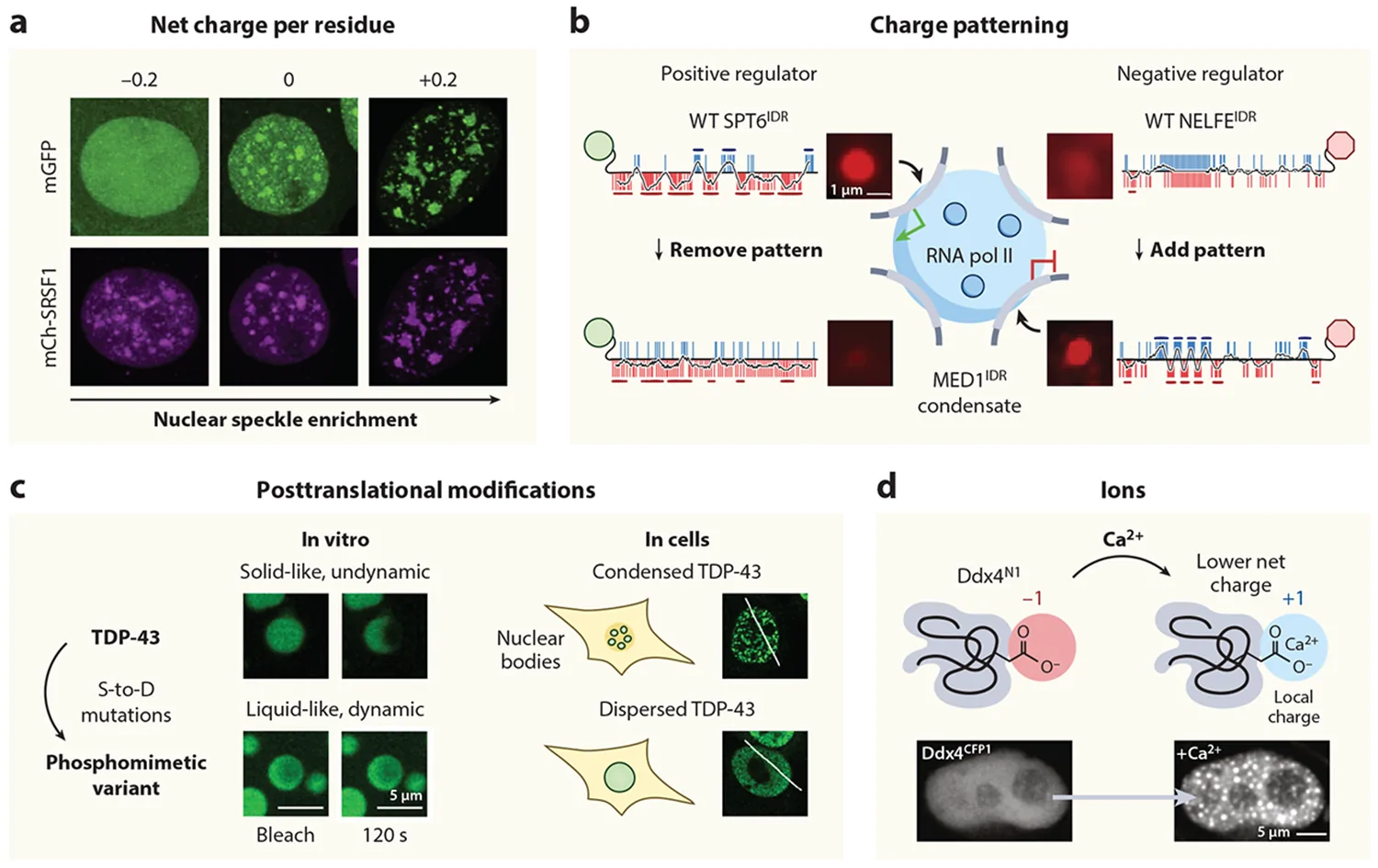
Parameters influencing the electrostatic interaction between proteins and PEs. (*a*) Increasing the net charge per residue can enhance nuclear speckle enrichment. (*b*) Charge patterning is important for selective partitioning and transcriptional activation in MED1 condensates. (*c*) Phosphomimetic substitutions enhanced fluidity of TDP-43 condensates in vitro and suppressed TDP-43 condensation and aggregation in cells. (*d*) Ions such as Ca^2+^ can bind with low affinity to negative residues in the disordered regions of Ddx4, effectively reducing the protein net charge and promoting phase separation in the cell. Abbreviations: PE, polyelectrolyte; TDP-43, TAR DNA-binding protein; WT, wild type. Panel *a* adapted from Reference 49. Panel *b* adapted from Reference 50. Panel *c* adapted from Reference 58 (CC BY 3.0). Panel *d* adapted from Reference 52 (CC BY 4.0).

Given that the building blocks of both proteins and PEs are often charged at physiological pH, electrostatic interactions play a central role in many biological examples of protein–PE complexation. Under physiological conditions, natural PEs such as RNA and DNA have high linear charge density with one negative charge per monomer from the phosphate backbone. In contrast, only 4 of the 20 amino acids that constitute proteins are charged at physiological pH. Consequently, proteins often have a variable net charge with a significantly lower charge density than that of typical biological PEs. These drastically different charge properties have led to systematic studies of how the net charge, surface charge, and charge patterning of both proteins and PEs affect protein–PE complexation and phase separation. For example, Greig et al. (49) found that the net charge of intrinsically disordered mixed-charge domains is a key determinant in nuclear speckle condensation, whereby increasing the negative charge abolishes speckle incorporation and increasing positive charge through arginine residues leads to greater condensation, possibly through complex coacervation with negatively charged protein regions and/or RNA (Figure 5a). Similarly, studies using engineered supercharged green fluorescent protein (GFP) variants have shown that net charge is one of the primary sequence determinants governing condensate formation with nucleic acids or disordered proteins like the Nephrin intracellular domain (3, 59, 60).

**Nuclear speckle:**

dynamic membraneless organelle found in the nucleus and involved in regulating gene expression, gene splicing, and RNA processing

**Complex coacervation:**

phase separation driven by electrostatic interactions, resulting in the formation of two liquid phases

The patterning of charge on proteins, and especially on IDRs, is another sequence determinant that can have an effect on protein–PE complexation. In particular, Lyons et al. (50) recently found that condensates composed of the largest IDR within the Mediator complex (MED1^IDR^) can activate transcription by selectively partitioning RNA polymerase II and positive allosteric regulators, such as SPT6, while excluding negative regulators, such as NELFE. Specifically, alternating blocks of charged amino acids on the SPT6 IDR are required for selective partitioning, and disruption of this charge pattern prevents incorporation of the protein into MED1^IDR^ condensates (Figure 5b). Similarly, the addition of a charge pattern to the NELFE IDR promotes partitioning into the condensate.

### 3.4. Posttranslational Modifications, Ions, and Solvent

Although the primary amino acid sequence of a protein may be fixed, posttranslational modifications (PTMs) such as phosphorylation and acetylation can reversibly alter charge and charge patterning to dynamically control protein–PE interactions and suppress or promote condensation (Figure 5c). For example, the ability of TDP-43 to form condensates appears to be influenced by various PTMs, including phosphorylation and acetylation. Using phosphomimetic substitutions, Gruijs da Silva et al. (58) found that C-terminal hyperphosphorylation leads to enhanced liquidity of TDP-43 condensates in vitro and suppresses TDP-43 condensation and aggregation in cells. Conversely, acetylation of TDP-43, particularly at lysine residues critical for RNA interactions, can disrupt its ability to bind RNA and promote the formation of insoluble TDP-43 aggregates (61).

Likewise, a range of intracellular ions can modulate the strength and nature of electrostatic protein–PE interactions, influencing the stability, dynamics, and composition of the condensates. For physiological concentrations of monovalent ions such as Na^+^ and K^+^ , electrostatic screening can be described by Debye–Hückel theory, while multivalent ions such as Ca^2+^ and Mg^2+^ can bind to charged residues and alter their attractive or repulsive interactions. One study observed Ca^2+^ ions binding with low affinity to the negatively charged residues in the disordered regions of the Ddx4 protein, reducing the protein’s net charge and promoting phase separation (52).

## 4. ENGINEERING SYNTHETIC PROTEIN–POLYELECTROLYTE CONDENSATES

Synthetic condensates, formed by LLPS of native or synthetic biomolecules, have been used to probe the behavior of endogenous biomolecular condensates (as described in Section 3, above) and to engineer new functionalities undiscovered in or unavailable to biological systems (62). The use of in vitro studies of binary or ternary protein–PE mixtures devoid of intracellular complexity has helped to provide a mechanistic understanding of protein–PE interactions governing condensate behavior in vivo. This fundamental understanding has, in turn, guided the design of synthetic condensates with potential for a range of cellular and noncellular applications.

Binary protein–PE or ternary protein–PE–PE synthetic condensates with phase behavior governed by a few parameters have been extensively studied to probe the equilibrium behavior of condensates. The use of simplified in vitro models allows the parameters that affect noncovalent interactions between the molecules forming condensates to be systematically varied. These parameters include pH (63), temperature (64), ionic strength (65), and relative (66) and net concentration (67) of the molecules undergoing phase separation. Although it is difficult to tune these parameters orthogonally in vivo, many functions of native condensates can be understood by probing their equilibrium thermodynamic behavior in vitro by individually tuning these parameters. Equilibrium properties that can be revealed using model synthetic condensates include composition, selective partitioning of small and large molecules, and formation or dissolution in response to external stimuli (40, 68).

Beyond revealing the equilibrium behavior of protein–PE condensates, synthetic condensates can recapitulate out-of-equilibrium features of biomolecular condensates. Control over properties of condensates, such as their number (69), cannot be achieved with simple thermodynamic equilibria but instead require active processes that either directly affect the phase-separating components or influence condensate behavior by modifying the local environment. In vitro systems that link droplet formation to chemical reactions, such as PTMs (see Section 3), can be used both to help elucidate the mechanisms underlying cellular control over condensate behavior under nonequilibrium conditions and, potentially, to create synthetic cells that sense and respond to their environment.

Protein engineering tools used to study endogenous condensate behavior (as highlighted in Section 3), such as single-amino-acid mutations or expression of recombinant protein variants, have similarly been used to explore the parameter space in synthetic models. By understanding the principles of protein–PE condensation, one can engineer synthetic systems with programmable or emergent functions for biomedical and synthetic biology applications, as described below.

### 4.1. Complex Coacervates as Synthetic Models for Equilibrium Biomolecular Condensates

Complex coacervates, formed by LLPS of oppositely charged PEs, have been well-characterized experimentally and theoretically since their discovery nearly a century ago (70) (see the sidebar titled A Brief History of Protein–Polyelectrolyte Condensation). Since then coacervates have been used as models to understand the equilibrium phase behavior of endogenous protein–PE condensates dominated by electrostatic interactions. Complex coacervates have a range of tunable parameters that govern the interaction strength between proteins and PEs, including macromolecule mixing ratio, net charge, and hydrophobicity, as well as solution ionic strength and pH.

**Figure 6:**
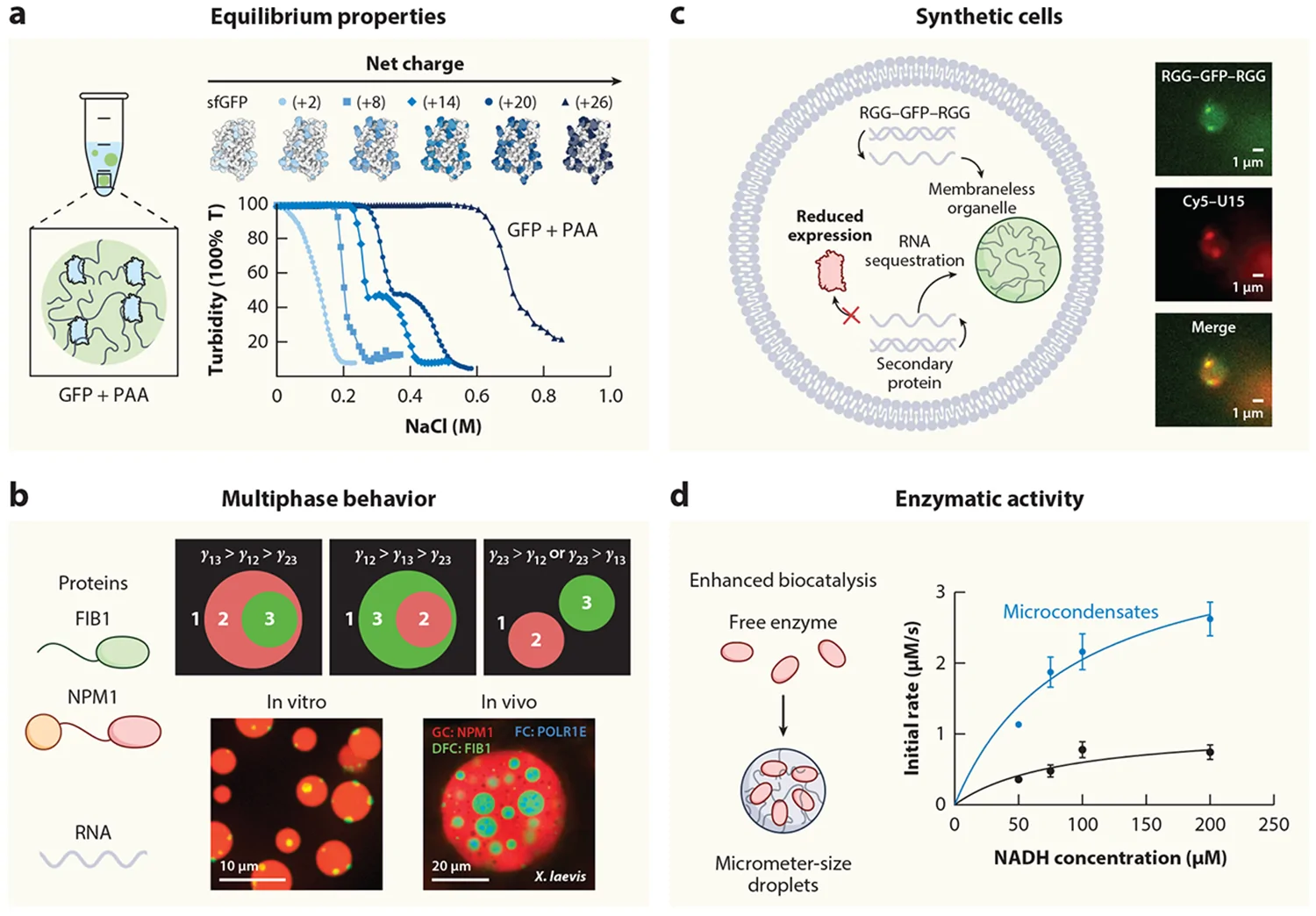
Examples of synthetic condensates. (*a*) Equilibrium properties of GFP–PAA coacervates such as salt resistance can be tuned by engineering the protein charge. Coacervates formed by GFP variants with higher net charge exhibit increased resistance to salt. (*b*) Possible thermodynamic scenarios for the formation of multiphase coacervates via mixing of two phase-separating proteins with RNA, thereby creating two immiscible dense phases with differences in surface tension. (*c*) In vitro demonstration of (*left*) multiphase architecture observed in (*right*) endogenous nucleoli using FIB1 (*green*) and NPM1 (*red*) proteins found within in vivo nucleoli. Formation of RGG–GFP–RGG protein condensates within liposomes interferes with the translation of a second protein by recruiting mRNA. Fluorescence microscopy images demonstrate the ability of RGG–GFP–RGG condensates to colocalize RNA within them. (*d*) Condensates formed by an NADH oxidase enzyme fused with intrinsically disordered regions from DEAD-box helicases show enhanced activity compared with the free enzyme in solution. Abbreviations: FIB1, fibrillarin 1; GFP, green fluorescent protein; NPM1, nucleophosmin 1; PAA, poly(acrylic acid). Panel *a* adapted with permission from Reference 91; copyright 2018 American Chemical Society. Panel *b* adapted with permission from Reference 93; copyright 2016 Elsevier. Panel *c* adapted with permission from Reference 94; copyright 2024 American Chemical Society. Panel *d* adapted with permission from Reference 95; copyright 2024 Springer Nature.

At the most basic level, complex coacervates have been used to determine the conditions under which proteins undergo phase separation or partition into condensates. Proteins require a minimum excess of either cationic or anionic charge, calculated on the basis of the charged amino acid residues at the given pH, to phase separate with oppositely charged polymers (90). Proteins with a higher net charge generally have higher encapsulation efficiencies and phase separate over a wider parameter space (91). Increasing the net charge of the protein via point mutations increases the resistance of the protein–PE coacervate to high salt concentrations but can also result in aggregate formation at low ionic strength as the protein–PE complexation is kinetically trapped (91) (Figure 6a). Proteins that do not undergo phase separation under physiological conditions can be engineered to phase separate by increasing their net charge either by chemical means (90) or by protein engineering approaches, such as site-directed mutagenesis (91) or fusion to charged sequences (92).

Alternatively, proteins can be encapsulated within polymer–polymer complex coacervates. By understanding the competition between the like-charged protein and polymer to phase separate with the oppositely charged polymer, one can tune the encapsulation efficiency of the protein within the two-polymer, three-component system (96). In this scenario, for the polymer of the same-sign charge as the protein, decreasing the overall charge on the polymer increases protein partitioning in the coacervate. However, the protein net charge and the macromolecular ratios are not sufficient to describe the encapsulation of proteins in the coacervate phase. Because of the relatively fixed geometry of globular proteins, the spatial distribution of charge on the protein surface also influences protein incorporation into ternary coacervates. Proteins with a less uniform distribution of charge have a region of higher charge density, known as a charge patch, and can more efficiently partition into coacervates but are also more sensitive to polymer chain length in comparison to proteins with more homogeneous charge distribution (96).

These relatively simple complex coacervate models recapitulate the formation of fluid-like dense droplets observed in many endogenous biomolecular condensates. However, natural condensates, such as nucleoli, can have more complex morphologies, with distinct phases existing within the dense phase that differ in composition (93). Multiphase coacervate assemblies have been used to probe the biophysical criteria for the formation of condensates with distinct internal organization (97). These multiphase coacervates can be created from three or more PEs, which can lead to the encapsulation of one coacervate phase within another coacervate phase of a different composition, suspended in a common dilute phase. Such multiphase assemblies, capable of partitioning small molecules and proteins, are equilibrium structures that arise from differences in surface tension between the individual coacervate phases and each coacervate phase with the dilute phase (98). The existence of two (or potentially more) distinct demixed regions within a single droplet arises from differences in effective interaction parameters, which are intrinsically linked to the macromolecule density in the coacervate phase. The rationale that differences in surface tension provide the basis for multiphase coacervate architecture, as explained by model protein and PE systems, can be extended to explain the three-layered condensate structure of the nucleolus. The proteins nucleophosmin 1 (NPM1) and fibrillarin 1 (FIB1) are major components of two of the layers (93). A study of the composition of in vitro synthetic condensates, obtained by mixing different ratios of NPM1 and F1B1 with and without rRNA, identified the conditions in the phase diagram that would lead to encapsulation of FIB1-rich condensates within NPM1-rich condensates similar to in vivo nucleoli composition (93) (Figure 6b). Demixing of the FIB1-rich condensate from the NPM1-rich condensate occurs because of differences in surface tension; the NPM1-rich phase is more hydrophilic than the FIB1-rich phase (93).

### 4.2. Out-of-Equilibrium Behavior of Synthetic Condensates

Research during the past century has provided a basic understanding of the equilibrium behavior of protein–PE condensates. However, cells are, by necessity, out of equilibrium, and any phase-separated bodies within cells will be affected by ongoing active processes. In a primary example, transcriptional condensates help regulate the process of RNA transcription. Condensates formed by phase separation of RNAs with transcriptional proteins in the Mediator complex control RNA transcription by a feedback mechanism in vitro and in cells (99). Electrostatic-based transcriptional condensate assembly is favorable at low RNA concentrations, and condensate formation increases the rate of RNA production by colocalizing the reactants. A high concentration of RNA then destabilizes the condensate, thereby slowing down transcription.

Therefore, given the importance of the nonequilibrium behavior of condensates in cells, many recent studies have focused on developing synthetic models that leverage knowledge of equilibrium phase behavior and the interdependence of phase separation and active processes such as chemical reactions. The development of synthetic condensates that recapitulate or account for out-of-equilibrium behavior could enable a physiological understanding and create new functional materials. For example, a recent study demonstrated that a synthetic condensate can regulate translation in a synthetic cell (94). The synthetic cell contained two plasmids: One encoded a fluorescent protein fused with intrinsically disordered domains known to form condensates (RGG–GFP–RGG), while the other expressed a test protein. The formation of RGG–GFP–RGG protein condensates suppressed the translation of the test protein as the transcribed mRNA partitioned into the condensates due to the presence of the RGG–RNA binding domain (Figure 6c).

Coupling of condensates and active processes can achieve more than simple acceleration or inhibition of chemical reactions. While equilibrium interactions between phase-separating proteins and RNA play a role in maintaining condensate fluidity and preventing aggregation, maintaining condensates out of equilibrium using energy-consuming processes also affects the material properties of condensates by slowing down protein aggregation. A study of the material properties of condensates formed between RNA and the ATPase Dhh1, a member of the DDX family, revealed that the addition of ATP enables enzymatic turnover and increases the fluidity of the condensates (100). These rheological measurements of synthetic condensates, both in and out of equilibrium, can lead to a fundamental understanding of how active processes tune condensate material properties, which, in turn, could be used to engineer ways to prevent disease-linked structural transitions and aging within native condensates (101).

In addition to the ability to provide insights into endogenous condensates, synthetic condensates serve as ideal platforms for the creation of synthetic cells. The high macromolecular concentration within the interior resembles the intracellular milieu, and the internal environment is compatible with biologically relevant substrates and enzymatic reactions. The condensate environment can either promote enzymatic activity by colocalizing enzymes and their substrates or inhibit activity by the selective partitioning of the enzyme or its substrate (Figure 6d). Theoretical models have shown that enhancement of enzyme activity within active condensates can enable size control (102) and division of drops (103), results that should inspire experimental validation with synthetic models (104). In addition to promoting enzymatic activity by increasing the local concentration of the reactants, the condensate environment can increase enzymatic activity by changing the affinity of the enzyme toward its substrate (e.g., a decrease in substrate *K*_m_) (105). Finally, the size of synthetic condensates can influence the kinetics of enzymes in the dense phase as a result of potential diffusion limitations at longer length scales and/or through more viscous environments (63, 95).

## 5. ENGINEERING PROTEIN–POLYELECTROLYTE INTERACTIONS FOR NANOSCALE ASSEMBLY

While the preceding sections have focused on protein–PE interactions driving macroscopic phase transitions that create micrometer- to millimeter-sized droplets, simple engineering of the PE can result in the formation of nanoscale assemblies instead. Interactions between proteins and block copolymer PEs can result in the formation of a range of nanoparticles, including vesicles (106-108), lamellae (109, 110), and micelles (111). The properties of the protein and PE components that form the nanoassembly not only affect the surface of the nanoparticle but also influence the assembly process itself and dictate the size, morphology, and stability of the assembled particle. Critically, the small size of nanoparticles (10–500 nm) and the resulting high surface area–to–volume ratio result in distinct physicochemical properties compared with those of bulk materials and give rise to several features that make them attractive for cellular applications. Below, we review efforts to engineer the protein and PE components to promote nanoparticle formation and stability so as to enable both in vitro and in vivo applications, largely in the area of intracellular protein delivery.

### 5.1. Protein–Polyelectrolyte Nanoparticles for Intracellular Delivery of Proteins

Perhaps the most significant application of protein–PE nanoassemblies is in the intracellular delivery of therapeutic proteins. While many protein therapies have shown great success in the clinic, they are limited by poor cellular membrane permeability, resulting in a limited ability to reach intracellular targets. Nanoscale carriers offer a potential remedy to this challenge, as they can both increase protein stability during formulation and circulation and increase intracellular delivery of the therapeutic protein cargo. A range of nanocarriers, comprising polymers, polysaccharides, or lipids, have been developed (115). However, the delivery vehicles created for hydrophobic small-molecule therapies, as well as those developed for high-charge-density nucleic acids, often struggle to effectively encapsulate and deliver active protein cargo, likely because of the hydrophilic and ampholytic nature of proteins. Nanoparticles formed via electrostatic interactions between proteins and PEs could fulfill the demands of a nanoscale intracellular protein delivery vehicle. In particular, the ability to tune the protein and PE components to control particle assembly and properties enables these self-assembled nanoparticles to meet the precise size, morphology, and stability requirements for efficient cellular uptake and protein release (115).

**Figure 7:**
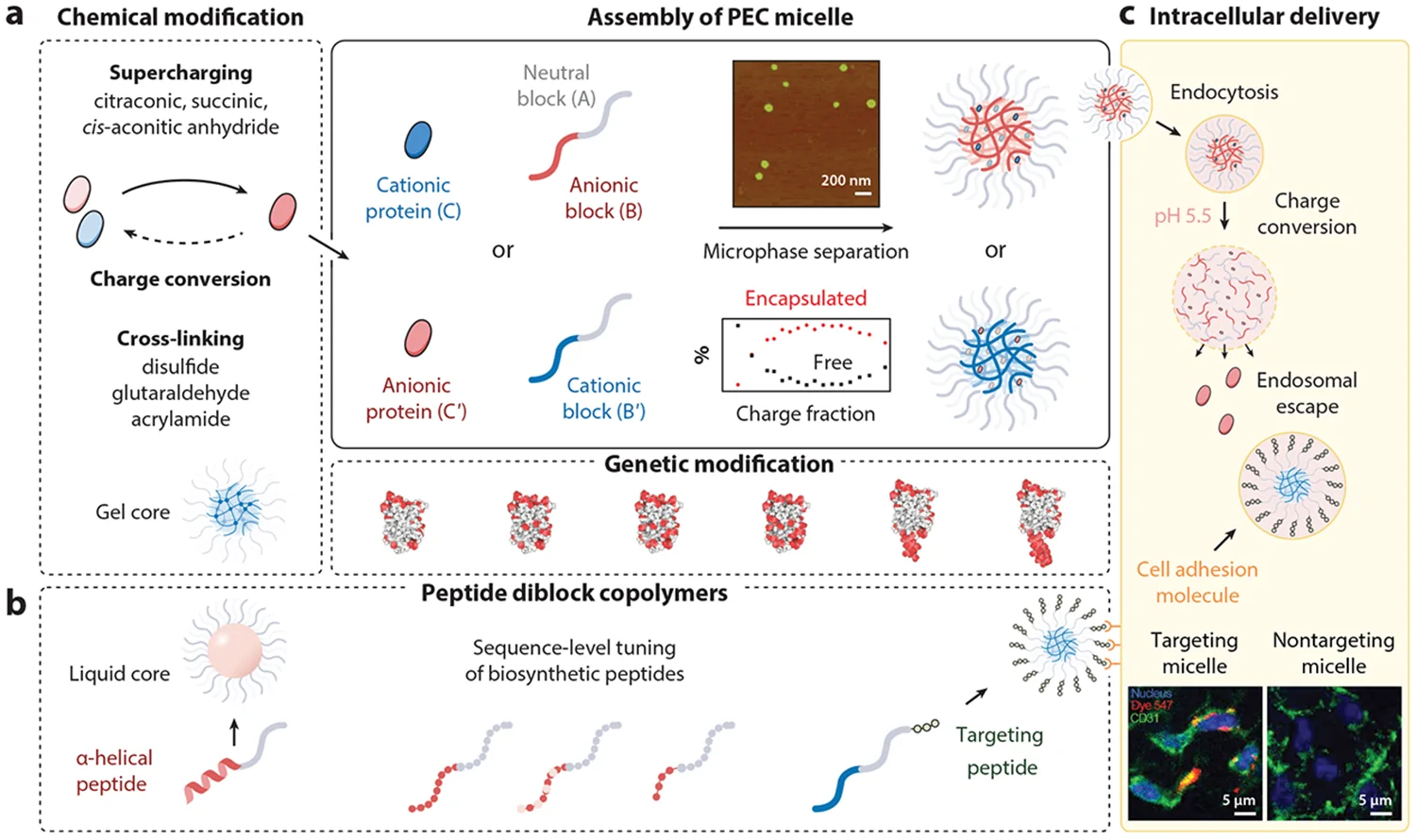
(*a*) Stable PEC micelles with proteins can be formed by chemical supercharging of the protein, chemical cross-linking of the core, or genetic modification of the protein cargo. Protein cargo encapsulation has been characterized by fluorescence correlation spectroscopy. (*b*) Peptide diblock copolymers for PEC micelle formation enable liquid-like cores, precise tuning, and cellular targeting. (*c*) Intracellular delivery of hydrophilic therapeutics, including proteins, by PEC micelles. Charge conversion by citraconic anhydride is reversed in the acidic endosome, triggering the disassembly of PEC micelles and endosomal escape. Targeting peptides displayed on the surface of PEC micelles facilitate cell-specific endocytosis. Abbreviation: PEC, polyelectrolyte complex. Panel *a* adapted with permission from Reference 113; copyright 2015 American Chemical Society. Atomic force microscopy image in panel *a* adapted with permission from Reference 112; copyright 2009 Wiley. Panel *c* adapted from Reference 114 (CC BY-NC-ND 4.0).

Among the best-studied protein–PE nanoassemblies are polyelectrolyte complex (PEC) micelles, also known as complex coacervate core micelles or polyion complex (PIC) micelles. PEC micelles form via interactions between a diblock copolymer, composed of a neutral hydrophilic block (A) and a charged block (B), and an oppositely charged PE (C). Complexes that form between the oppositely charged PEs, B and C, are stabilized to coarsening and shielded from solvent interactions by the neutral block, A (Figure 7a). This process results in microphase separation, leading to the formation of micelles of a consistent morphology and size. Of interest here are micelles where the oppositely charged PE (C) is a protein, as well as those where both components of the PEC micelle (A and B) are proteins.

Following an initial report by Harada & Kataoka (116) of PEC micelle formation between polypeptide-based block copolymers, interest in their use for therapeutic delivery steadily increased. PEC micelles are attractive for delivery applications because of the ability to control their size and morphology as well as to incorporate hydrophilic cargo, including nucleic acids and proteins (117-123). Given that the micelles are held together by dynamic electrostatic interactions, they can respond to environmental changes or stimuli, enabling selective release of cargo (124-126). In addition to the responsive core, the core–shell structure offers the opportunity to design an inert shell to minimize immune recognition or to target specific cell surface markers during in vivo delivery (127).

Below, we discuss recent efforts to engineer PEC micelles for efficient cellular delivery of protein cargo. First, we review studies aiming to engineer the protein cargo (C) to improve micelle formation, stability, and/or function (Figure 7a). We cover both bioconjugation and genetic approaches to modify the protein to promote micelle formation and, ultimately, intracellular protein delivery (Table 2). We then describe recent efforts to engineer intrinsically disordered protein block copolymers to replace the synthetic (AB) block copolymers initially used to form PEC micelles (Figure 7b). Transitioning to fully protein-based PEC micelles will enable the precise design of both the physiochemical and biological properties via engineering of the protein and PE components.

### 5.2. Engineering Globular Protein Cargo for Polyelectrolyte Complex Micelle Formation

In order to create PEC micelles for protein delivery, the protein cargo must be efficiently incorporated into the micelle core via electrostatic interactions. However, as discussed above, proteins have relatively low charge density (only 4 of the 20 amino acids carry net charge at physiological pH) and are polyampholytes (the four charged monomers are split evenly between positive and negative charge). As a result, the protein cargo is often unable to form, or stably partition into, PEC micelles at physiological pH and ionic strength. Several approaches to engineering the protein cargo have been developed to address this limitation: (*a*) chemical modification of the primary amine of lysine residues to increase the net negative charge, (*b*) chemical cross-linking of the PEC micelle core to improve stability, and (*c*) genetic modification to increase the net charge. Each approach has advantages and disadvantages, as discussed below.

Proteins can be modified chemically, typically via reaction of lysines with a cyclic anhydride, to increase the net charge without altering the primary amino acid sequence (Figure 7a). A primary advantage of this approach is that some modification methods are fully reversible with modifications cleaved either chemically or enzymatically. An investigation of the relationship between the degree of chemical modification and PEC micelle formation and stability found that increasing the charge density on the protein, via chemical modification with succinic anhydride, improves the stability of PEC micelles (90). While modification with succinic anhydride is not reversible, simply switching the cyclic anhydride to citraconic anhydride renders the modification pH sensitive (112). This approach has been used to completely convert surface lysine residues so as to create pH-responsive micelles that dissociate in acidified endosomes during intracellular delivery. PEC micelles created with a charge-converted immunoglobulin G formed stable micelles that successfully delivered active antibody to the nuclear envelope following endocytosis, cleavage of the modification, micelle disassembly, and endosomal escape (111, 130, 131) (Figure 7c). While this approach has been successful for several proteins, a remaining challenge is the lack of site specificity of the modification. This can result in inactivation or unfolding of sensitive protein cargo, ultimately preventing broad application of this approach.

As an alternative, the protein (as well as the diblock copolymer) component can be chemically modified following PEC micelle assembly. In this case, electrostatic interactions between the protein and PE promote self-assembly at low ionic strength, and then chemical cross-linking of the preformed micelle can stabilize the nanoparticle at high ionic strength. Several core cross-linking methods with protein cargo have been demonstrated; they include disulfide-bond formation between two free thiols (111, 131) and imine-bond formation with a dialdehyde (132). This general approach has been used to create PEC micelle–templated nanogels as well as PICsomes (106, 107) and electrostatically templated single-enzyme nanoparticles (133, 134).

Finally, genetic engineering approaches enable the precise modification of proteins to tune their net charge. Site-directed mutagenesis can be used to increase or even reverse protein net charge, typically by mutating charged or polar surface residues, in a process known as supercharging (Figure 7a). For example, PEC micelles formed with a model GFP showed a linear correlation between the protein net charge and stability to increased solution ionic strength (128). The precision of genetic engineering enables the introduction of more extensive changes to the charge, too; instead of isotropically distributing charge on the protein surface, specific patches of localized charge can be introduced on the surface or appended as a charge-dense peptide tag (135). The introduction of a charged peptide tag onto GFP enables its incorporation into PEC micelles, although micelles formed with the charged peptide on GFP show lower salt stability than do micelles formed following isotropic supercharging. However, the localized charge results in superior intracellular delivery of the GFP cargo (128). A similar C-terminal peptide tag was also fused to the enzyme CotA, which successfully incorporated the protein into PEC micelles (136). Fluorescence correlation spectroscopy measurements showed that enzymes with the peptide tag form more PEC micelles with fewer CotA molecules per micelle, as would be expected to maintain charge neutrality (136).

### 5.3. Engineering Proteins as Polyelectrolyte Diblock Copolymers

In addition to the protein cargo in the core, recent efforts have focused on the use of protein engineering to create PE diblock copolymers to displace the synthetic polymer component in PEC micelles. The initial report of PEC micelles from Harada & Kataoka (116) used poly(l-lysine) and poly(α, β–aspartic acid) polypeptides for the coacervate core and poly(ethylene glycol) (PEG) for the neutral corona. Since then, many studies have also used synthetic block copolymers containing a charged polypeptide block to facilitate biocompatibility (124). Recent reports have demonstrated additional advantages of including polypeptide components in the PE block copolymer. For example, the use of a polypeptide with strong α-helical content resulted in PEC micelles that maintained a liquid-like coacervate core and promoted coacervate stability to increased ionic strength, both of which are key requirements for delivery of encapsulated biomolecules (137). Moving to in vivo delivery, fusion of a targeting peptide to PEG–poly(l-lysine) created peptide-decorated PEC micelles that successfully delivered nucleic acid cargo to inflamed tissue (114, 138) (Figure 7c). While these examples still used synthetic polypeptides chemically linked to synthetic polymers, more recent research has begun to evaluate polypeptide block copolymers that can be designed at the genetic level and produced biosynthetically. This switch to biosynthetic protein PE block copolymers offers the distinct advantage of being able to directly program in different features at the genetic level, from targeting to endosomal escape to protein complexation.

While much of the discussion so far has focused on the use of protein engineering tools to modify native protein sequences, these tools, when combined with low-cost DNA synthesis, can also be used to design proteins, or protein chimeras, from scratch. This approach has been employed for more than three decades to create simplified versions of complex natural proteins, often by using repeats based on consensus sequences (139-141). Perhaps the best-studied of these designed proteins are a class known as elastin-like polypeptides (ELPs), which contain repeats of the pentapeptide VPGXG (where X is a guest residue and can be nearly any of the 20 native amino acids). The ELP sequences are disordered and soluble at low temperatures, but they phase separate above a lower critical solution temperature (LCST). Critically, this LCST can be tuned at the protein sequence level by varying the guest residue as well as the length of the overall polypeptide. Recent research has shown that a neutral ELP, containing valine as the guest residue, can be fused to a charged polypeptide domain to promote PEC micelle formation (65). Our group has demonstrated that ELP fusion with an anionic polypeptide, derived from the human protein prothymosin α, creates a biosynthetic PE that can complex with cationic globular proteins to create PEC micelles (64) (Figure 7b). The micelles are sensitive to the ionic strength of the solution and the temperature as a result of the electrostatic interactions between the charged proteins and the LCST behavior of the ELP block, respectively. Similarly, the oppositely charged ELP diblock copolymers (VPGKG)_32_-(VPGVG)_64_ and (VPGEG)_32_-(VPGVG)_64_ form PEC micelles at low salt concentrations. However, the PEC micelle inverts to create an amphiphilic micelle at high ionic strength and high temperature (142). These reports demonstrate the potential of biosynthetic polypeptide PEs for the creation of well-defined and responsive nanoassemblies. Future studies should seek to evaluate their biocompatibility, tunability, and intracellular cargo delivery.

## 6. CONCLUSIONS

Over the last few decades, protein engineering has become a routine and accessible technique, which has led to an improved understanding of protein structure–function relationships and the development of protein variants with tailored properties. The studies described in this review highlight instances where protein engineering has been used in interesting ways to control protein–PE interactions, whether through structure, charge, charge patterning, or environmental conditions. By discussing the interactions that drive complexes at the single-amino-acid level, in the context of both endogenous and synthetic phase-separated condensates and nanoscale assemblies, we have aimed to interrogate the fundamental principles that underlie these complexes and draw parallels between these fields. The use of protein engineering strategies to control or design protein–PE interactions currently appears to hold the greatest biomedical promise in terms of drug delivery, with protein–polymer micelles offering the potential for targeted delivery. Within the biomolecular condensate field, a rapidly growing body of work is pinpointing the role of specific interactions. Currently, much of this research is reductionist, focusing on individual proteins. Scaling this understanding to the system level could revolutionize our understanding of the cell. In particular, exciting emerging research on nonequilibrium condensate systems could open up entirely new ways to influence biochemical pathways in vivo. While the research presented in this review has laid the groundwork for the design of protein–PE interactions for a range of applications, outstanding questions remain to be addressed and are ripe for future exploration (see the next section).

## 7. FUTURE ISSUES

Most research to date has focused on tuning charge–charge interactions between proteins and PEs. However, as discussed in Section 2, proteins engage in a wide range of noncovalent interactions in water, the most important of which are the quadrupolar, hydrogen-bonding, π–π, and hydrophobic interactions. The role of these interactions in the formation, properties, and function of protein–PE complexes should not be ignored and is a ripe area for future exploration (36).

Water mediates interactions between proteins and PEs. This is best understood at the single protein–nucleic acid complex level (143). This role has begun to be investigated in mesoscale assemblies, and interesting results have emerged experimentally from the use of terahertz spectroscopy (144) and computationally from atomistic molecular dynamics simulations (145). Understanding how protein mutations affect water arrangement, and how that leads to changes in protein interactions, is an interesting area of emerging research.

Without a complete understanding of how amino acid mutations affect the interactome, fully rational design is not possible. Deep mutational scanning approaches can be used to screen a vast array of protein mutations. Several ways to produce libraries of genetic mutants exist, but there are fewer methods for high-throughput screening of how these mutants affect interactions with PEs. Combining existing methods for mutant library generation with phase-separation assays could provide a high-throughput way to screen for mutations that alter protein–nucleic acid interactions in cells or protein–PE interactions in vitro.

While significant progress has been made in the development of protein delivery vehicles, continued efforts are needed to improve the biocompatibility of these drug delivery systems. One promising approach utilizes combinatorial synthesis, high-throughput screening, and machine learning to identify the design criteria for polymeric delivery agents (146). An alternative approach looks to biology for inspiration and displaces the synthetic polymers used to create nanoassemblies with engineered disordered protein polymers (147). Continued development of each of these approaches, as well as the convergence of high-throughput methods and engineered protein polymers, offers great promise for the development of biofunctional nanoscale protein–PE complexes.

## Figures and Tables

**Figure 2:**
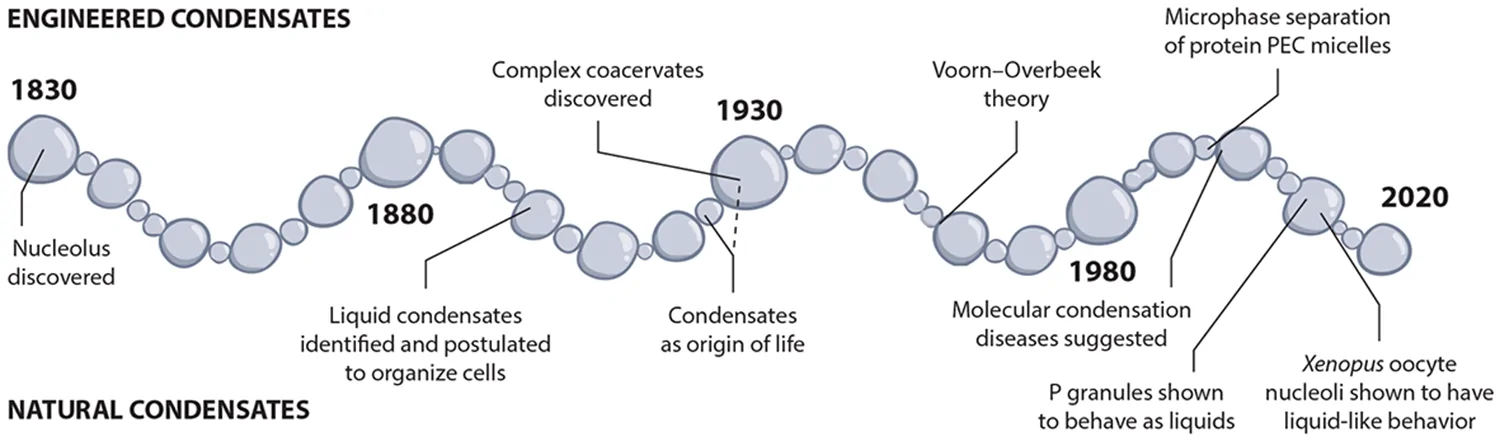
Timeline of condensate events. Abbreviation: PEC, polyelectrolyte complex.

**Table 1:** Important parameters of proteins and PEs in biomolecular condensates

Parameter	Protein	PE	Condensate	Reference
Protein structure	G3BP1	RNA	Stress granule	46
PE structure	Whi3	mRNA	Whi3 condensate	47
Surface charge	CAPRIN1	RNA	Stress granules/P bodies/mRNA granules	48
Net charge, charge density	snRNP70, CPSF6	RNA	Nuclear speckles	49
Charge patterning	MED1IDR	RNA	Transcriptional condensate	50
PTMs	TDP-43	RNA	Stress granule, nuclear bodies	51
Ions	Ddx4	RNA	D bodies	52

Abbreviations: PE, polyelectrolyte; PTM, posttranslational modification; RNP, ribonucleoprotein.

**Table 2:** Examples of engineered protein–PE interactions in the design of protein-containing micelles

Protein	PE	Modification parameter	Application	Reference
GFP	ELP–prothymosin α fusion^a^	Block length, charge density	No data	64
GFP^a^	POEGMA-b-qP4VP	Isotropic charges, charged tags	Intracellular delivery	128
CotA laccase^a^	PM2VP-b-PEO	Isotropic charges, charged tags	No data	129
α-Chymotrypsinogen,^a^ lysozyme,^a^ myoglobin,^a^ RNase A^a^	POEGMA-b-qP4VP	Succinic anhydride supercharging	No data	90
Cytochrome *c*^a^	PEG–pAsp(DET)	Citraconic anhydride, *cis*-aconitic anhydride, succinic anhydride supercharging and charge conversion	Intracellular delivery	112
Immunoglobulin G^a^	PEG–pAsp(DET)	Citraconic anhydride supercharging	Intracellular delivery	130
3D6 Fab,^a^ 6H4 Fab^a^	PEG–poly(Lys)	Citraconic anhydride supercharging, disulfide cross-linking	Brain delivery	131, 111
Glucose oxidase	PEG-b-p(Boc-METMA)^a^	Glutaraldehyde cross-linking	Intracellular delivery	132

a Modified components.

Abbreviations: ELP, elastin-like polypeptide; GFP, green fluorescent protein; PE, polyelectrolyte; PEG-b-p(Boc-METMA), poly(ethylene glycol)-*block*-poly[*tert*-butyloxy-l-methionine-(2-methacryloylethyl)ester]; PEG–pAsp(DET), poly(ethylene glycol)-*block*-poly{*N*-[*N* ′ -(2-aminoethyl)-2-aminoethyl] aspartamide}; PEG–poly(Lys), poly(ethylene glycol)-*block*-poly(l-lysine); PM2VP-b-PEO, poly(*N*-methyl 2-vinyl pyridinium)-*block*-poly(ethylene oxide); POEGMA-b-qP4VP, poly(oligoethylene glycol methacrylate)-*block*-poly(*N*-methyl 4-vinyl pyridinium iodide).

## A BRIEF HISTORY OF PROTEIN–POLYELECTROLYTE CONDENSATION

One of the earliest examples of protein–PE condensates comes from cell biology, with the discovery of the nucleolus in 1830 (71). We now know that this organelle is membraneless and that its formation is governed, at least in part, by protein–RNA interactions. The first suggestion that organelles such as the nucleolus can behave in a liquid-like manner was put forward in 1899 by Edmund Wilson (72). Wilson also hypothesized that this behavior could be a governing principle of cellular organization. Expanding on the central role of biological emulsions, Alexander Oparin (73) postulated the existence of biomolecular phase separation in 1924 as a potential mechanism underlying the origin of life, and Haldane (74) expanded upon this idea in 1929 in his research on the origin of life.

Following these early observations (and likely as a result of the introduction of cell fixation, which introduces a bias toward the study of membrane-bound organelles), phase separation driven by condensation of proteins and biological PEs was largely overlooked as a dominant process for cellular organization (75). Nearly a century after the first observations of the nucleolus, Bungenberg de Jong & Kruyt (70) demonstrated in vitro phase separation of proteins and polysaccharides. Their experiments led to the coining of the phrase complex coacervation and underpinned the first theoretical frameworks describing electrostatically driven coacervation, put forward by Michaeli et al. (76) and Overbeek & Voorn (77). The advent of reliable methods for polymer synthesis prompted a shift away from studies of biological materials and toward synthetic systems. These developments led to our current understanding of complex coacervation and to the design of a range of PE-based condensates, such as droplets and complex coacervate core micelles (78).

The pendulum swung back to biological protein–PE condensates following the 2009 discovery by Brangwynne et al. (79) that RNA and RNA-binding protein P granules behave as liquid droplets inside *Caenorhabditis elegans* embryos. Two years later, another study demonstrated this liquid-like behavior for frog oocyte nucleoli (80). Following these landmark reports, investigators identified many other membraneless organelles that form via the condensation of proteins, often with biological PEs (DNA and RNA) (81), including SGs (82, 83), P granules (57, 84), and yeast ribonucleoprotein granules (85). At the same time, several groups reported that distinct liquid assemblies can form via the expression of engineered proteins, creating synthetic condensates in cells (86, 87).

This resurgence of interest in protein condensation as an organizing principle in cells has led to renewed interest in complex coacervates as cellular mimics. Much of the theory developed to describe complex coacervates has been applied to the description of biomolecular condensates (88), and reductionist polymer systems have been used to provide insights into or recapitulate biological observations (68, 89) (Figure 2).
